# Brief, low frequency stimulation of rat peripheral C-fibres evokes prolonged microglial-induced central sensitization in adults but not in neonates

**DOI:** 10.1016/j.pain.2009.03.022

**Published:** 2009-07

**Authors:** Gareth J. Hathway, David Vega-Avelaira, Andrew Moss, Rachel Ingram, Maria Fitzgerald

**Affiliations:** UCL Department of Neuroscience, Physiology & Pharmacology, University College London, Gower Street, London WC1E 6BT, UK

**Keywords:** Rat, Neonate, Central sensitization, Microglia, Sciatic

## Abstract

The sensitization of spinal dorsal horn neurones leads to prolonged enhancement of pain behaviour and can be evoked by intense C-fibre stimulation, tissue inflammation and peripheral nerve injury. Activation of central immune cells plays a key role in establishing pain hypersensitivity but the exact nature of the afferent input that triggers the activation of microglia and other glial cells within the CNS, remains unclear. Here intense but non-damaging, electrical stimulation of intact adult rat C-fibres for 5 min at 10 Hz induced central sensitization characterized by significant decreases in mechanical withdrawal thresholds 3, 24 and 48 h later. This maintained (>3 h) hypersensitivity was not observed following topical skin application of capsaicin. C-fibre evoked sensitization was accompanied by significant microglial activation, shown by increased Iba-1 immunoreactivity throughout the dorsal horn at 24 and 48 h and significant upregulation of markers of microglial activation: IL-6 and Mcp-1 at 3 h and Mmp3, CSF-1 and CD163 at 24 and 48 h. C-fibre stimulation caused no nerve damage at ultrastructural and molecular levels. Lower intensity stimulation that did not activate C-fibres or sham stimulation did not increase Iba-1 immunoreactivity or induce behavioural sensitivity. Pre-treatment with minocycline (40 mg/kg, i.p.) prevented the C-fibre evoked sensitization and microglial activation. Identical C-fibre stimulation in 10-day old rat pups failed to activate microglia or change behaviour. These results demonstrate that a brief period of low frequency C-fibre stimulation, in the absence of nerve damage, is sufficient to activate microglia resulting in behavioural hyperalgesia.

## 1 Introduction

Prolonged alterations in sensory processing occur following peripheral inflammatory or neuropathic injury. These changes are not exclusively determined by peripheral sensory afferent activity, but also reflect modifications of the central processing of sensory information [61,62]. Nociresponsive networks in the dorsal horn are dynamic and can be profoundly altered by intense peripheral sensory stimulation leading to the induction of the phenomenon of central sensitization, a state where the response to noxious and innocuous stimuli is greatly enhanced [35,61]. Injury-induced central sensitization in the spinal dorsal horn is thought to underpin much of the allodynia and hyperalgesia that characterize acute and chronic pain.

The decrease in pain thresholds that characterize central sensitization is associated with an increase in the responsiveness of dorsal horn neurons and alterations in gene expression [64]. Stimuli that can produce central sensitization include tissue inflammation and nerve injury [12,21,26] but the key to central sensitization is that it requires intense or repetitive C-fibre nociceptor stimulation [20,54,65]. The prolonged LTP evoked in lamina I cells by both low and high frequency C-fibre stimulation is likely to be an important mechanism underlying the synaptic potentiation of stimulated afferents [48,51,54] along with a wider heterosynaptic facilitation of the NMDA receptor by convergent intracellular biochemical cascades in dorsal horn neurons [5,24,49].

It is becoming increasingly evident that the cellular changes associated with central sensitization are not restricted to neurons, and that non-neuronal cell types, particularly immune cells play a key role in the induction and maintenance of prolonged states of pain hypersensitivity [10,52,58]. Of particular interest are microglia. These are the primary immunocompetent cell type within the CNS serving a major role in the immune response to tissue injury or infection [28,60]. Exogenous application of activated microglia into the dorsal horn results in reduced sensory thresholds similar to those associated with central sensitization [39,58]. In pathological pain states, such as Tumour necrosis funtor-α following peripheral nerve injury, the involvement of microglia has been clearly demonstrated [7,16] and proinflammatory cytokines released by microglia have recently been shown to cause central sensitization and hyperalgesia by increasing excitatory synaptic transmission in dorsal horn cells [25].

While the link between tissue injury, microglial activation and hyperalgesia is strong, the nature of the afferent signals that trigger microglial activation is still not clear [36]. Identifying such factors might provide important targets for the treatment of chronic pain. We have tested whether direct stimulation of C-fibres in uninjured animals, at intensities known to cause central sensitization, is sufficient to cause microglial activation in the dorsal horn.

## 2 Methods

All animal procedures were licensed by the UK Home Office and performed in accordance with the Animals (Scientific Procedures) Act 1986. Animals (Sprague–Dawley rats) were housed in cages of six age-matched animals (adult males) or alongside their mothers and littermates (neonates) with free access to food and water. The room in which animals were housed had a 12-h light/dark cycle.

### 2.1 Adult and P10 (postnatal day 10) electrical stimulation protocol

Rats were anaesthetized with isoflurane (Abbott Animal Health, Queensborough, UK (1.5%)) and oxygen and body temperature maintained using a homeothermic-heated blanket (Harvard Apparatus, Kent, UK). Surgery was performed in sterile conditions, in a Bassaire P4VF (Southampton, UK) positive pressure hood using aseptic techniques. The left sciatic nerve was exposed via an incision through the thigh, the overlying muscle was moved aside using blunt dissection and the nerve dissected free of perineural membranes. The exposed nerve was electrically isolated from the surrounding muscle and other tissues by placing a small piece of plastic sheet under the nerve. Two silver wire electrodes were placed under the exposed sciatic nerve. Care was taken to ensure that the electrodes were only in contact with the nerve and that the sciatic nerve was never stretched. Trains of electrical stimuli were applied for 5 min at 10 Hz at different pulse widths and intensities, to recruit the following afferent fibre groups:- Aβ and Aδ fibres (150 μs, 5 mA,) or Aβ, Aδ and C-fibres (500 μs, 10 mA) as described elsewhere [32,63]. These two experimental groups of rats were labeled the “Aβ/Aδ” and “C” groups. A sham-operated group that underwent surgery and electrode placement but did not receive electrical stimulation was also included. Stimuli were generated using a Neurolog (Digitimer, Welwyn Garden City, UK) NL300 pulse generator, an NL510 pulse buffer and an NL800 stimulus isolator. Following stimulation, electrodes were carefully removed, the muscle and skin sutured using 5/0 Mersilk (Ethicon, Edinburgh, UK) and the animals returned to their home cage to recover with free access to food and water. Identical procedures were followed in both adults and the P10 group.

### 2.2 Capsaicin stimulation protocol

Adult rats were anaesthetized as above and 20 μg of capsaicin (8-methyl-*N*-vanillyl-6-nonamide) or 0.9% saline was injected into the plantar foot pad of one hindpaw in a volume of 10 μL. Capsaicin was prepared in polyxyethylene (20) sorbitan (Tween 80) saline vehicle as described previously [14] and injected using a 28G needle. Mechanical thresholds were established before capsaicin application and again 3, 24 and 48 h post capsaicin, using methods described below. Following the 48-h time point animals were killed and tissue processed for immunohistochemical evaluation of Iba-1 immunoreactivity (see below).

### 2.3 Minocycline inhibition of microglia activation

Adult rats were pre-emptively treated with minocycline (40 mg/kg; Sigma UK) i.p. 1 h prior to sciatic nerve stimulation (C-fibre strength or sham) and then every 24 h until the animals were killed and the spinal cord collected for Iba-1 immunohistochemical analysis (see below). Mechanical withdrawal thresholds were measured before stimulation and then 3, 24, and 48 h post stimulation as described below.

### 2.4 Immunohistochemistry

Adult and P10 rats were given an overdose of pentobarbitone [100 mg/mL] and transcardially perfused with cool heparinised saline followed by cool 4% paraformaldehyde. The lumbar spinal cord was removed, post-fixed (4% paraformaldehyde solution) and stored in 30% sucrose in 0.1 M phosphate buffer/0.02% sodium azide solution at 4 °C. Immunohistochemical staining was performed on 40 μm free-floating cryosections of L3/L4/L5 spinal cord. For Iba-1, the sections were blocked for 1hr in TTBS (0.05 M Tris saline, pH7.4/0.3% Triton-X100) containing 1% normal goat serum (NGS) at room temperature (RT). Sections were then incubated at 4 °C for 72 h with rabbit α-Iba-1 (Wako, Japan) diluted 1:2000 in TTBS. Three 10 min washes in 0.1 M phosphate buffer (PB) were carried out between all subsequent steps. Sections were incubated for 2 h at room temperature with Alexa 488 conjugated goat α-rabbit secondary antibody (Molecular probes, Oregon) diluted 1:200 in TTBS. Iba-1 immunostaining was analysed by measuring the intensity of fluorescence within a defined region of the dorsal horn using MCID-1 software (London UK). The L3-5 dorsal horn was divided into a medial, middle and lateral third in the dorsoventral plane and fluorescence intensity measured medially and laterally both ipsi and contralateral to sciatic nerve stimulation. Contralateral intensity was treated as basal and intensity changes in the ipsilateral side calculated as a percentage of contralateral values. Statistical analyses were performed using one-way ANOVA with the Bonferroni post-test.

### 2.5 Sciatic nerve ultra-thin section microscopy

Three days following sciatic stimulation adult rats were perfused transcardially with 4% paraformaldehyde 2% gluteraldehyde in 0.1 M PB. Tissue was post fixed for a minimum of 72 h in fixative and embedded in Epon. Sections were cut through the stimulation site at 0.5 μm and stained with toluidine blue. Sections were extensively examined under the light microscope for signs of axonal degeneration, leukocyte infiltration and shrunken or swollen axons.

### 2.6 Behavioural testing

Behavioural testing was performed in control rats (sham-operated or control saline intra-plantar injection), and experimental rats (sciatic nerve C-fibre stimulation groups and intra-plantar capsaicin group) in both adult and P10 rat pups. Basal mechanical withdrawal thresholds were determined in rats at least one day prior to the test procedure to habituate them to the room, the investigator and the procedure. Thresholds were tested again immediately before anaesthetizing and then at 3, 24 and 48 h post-stimulation. Flexion withdrawal reflex thresholds were established in both the groups to punctate mechanical stimulation of the plantar surface of the hindpaw using calibrated von Frey filaments (VF) that exert a reproducible stimulus strength in grams. (Stoelting, Woodvale, IL). Filaments were applied sequentially to the plantar surface of the hind paw 10 times at intervals of 1 s. Response threshold was defined as the VF filament which produced reflex paw withdrawal in 5 of 10 applications.

To assess the ability of C-fibre strength electrical stimulation to induce central sensitization over the 48 h duration of the study, repeated measures two-way ANOVA’s were performed between sham, C-fibre (ipsilateral and contralateral mechanical withdrawal thresholds as separate groups) and capsaicin-stimulated groups. Where ANOVA’s were significantly different (*P* < 0.05) the Bonferroni post-tests were performed to determine the degree of significance. The effect of minocycline upon behavioural responses 24 h after sciatic nerve C-fibre stimulation was tested using a Student’s *t*-Test.

### 2.7 RNA extraction

Fresh tissue was collected 3, 24 and 48 h after C-fibre stimulation or sham surgery (*n* = 4 animals per experimental group). Rats were sacrificed with 0.1 mL of Euthanal^®^, the ipsilateral side of the dorsal horn was snap frozen on liquid nitrogen and stored at −80 °C until RNA extraction. RNA was extracted using Trizol^®^ reagent (Invitrogen^®^) and QIAshredder column (Qiagen^®^) for homogenisation according to manufacturer’s protocols. Additionally, the RNA was cleaned by an RNA purification column (Qiagen^®^) and a final volume of 20 μL was obtained according to manufacturer protocol. The amount of RNA was measured with a NanoDrop ND-1000 Spectrophotometer (NanoDrop Technologies, Inc.). Then, the RNA was stored at −80 °C until cDNA synthesis. For cDNA synthesis, 2 μg of RNA and 100 pmol/μL of the T7(dT)24 primer (5′-GGCCAGTGAATTGTAATACGACTCACTATAGGGAGGCGGTTTTTTTTTTTTTTTTTTTTTTTT-3′) were combined for annealing at 55 °C for 5 min and chilled on ice for 2 min. Then, the SuperScript III kit (Invitrogen^®^) was added according to manufacturer indications to synthesize the cDNA.

### 2.8 Quantitative real-time PCR

Specific primers for microglial markers were designed and synthesized by Sigma-Genosys (Supplementary Table I). An SYBR green-based system (SIGMA^®^) was used for amplification and detection. 1 μL of cDNA product per sample, 12.5 μL of SYBR^®^ Green JumpStart™ Taq ReadyMix™ for quantitative PCR and 200 nM/primer was used for cocktail reaction. The amplification protocol was: step 1 at 95 °C for 5 min, step 2 at 95 °C for 30 min, step 3 at 60 °C for 30 min, step 4 at 70 °C for 30 min, step 5 plate reading, 39 cycles from step 2 to 4 and a final melting curve from 60 to 90 °C with reading every 0.5 °C and 1 min hold. The reaction plate was then placed in a DNA Engine Pelter thermal cycler with a Chromo 4 real-time PCR detector (Bio-Rad^®^). The amplification process was monitored with Opticon Monitor v3.1.32 software (MJ Genework Inc.).

The reported fluorescence was obtained with a standard curve of serial dilutions of a cDNA calibrant. We performed a standard curve per gene using the corresponding specific primers. We set up a ‘threshold’ in the lineal range right above the baseline noise in order to obtain an *r*^2^ < 0.999 in the regression curve to accurately determine the fluorescence.

The DNA products from the qPCRs were sequenced on an AB PRISM 3100-Avant automated DNA capillary sequencer (AB Applied Biosystems, Lingley House, 120, Birchwood Boulevaid, Warrington) using BigDye Terminator v1.1 Cycle Sequencing chemistry (AB Applied Biosystems). Sequencing reactions were performed and precipitated in accordance with the manufacturers instructions using the same sense or forward primer that was used for the qPCR amplification (Supplementary Table I). The reactions were resolved on a 50 cm standard sequencing capillary, running optimal 3100 POP-6TM polymer (AB Applied Biosystems). Sequence data were extracted and analysed using AB PRISM 3100-Avant Data Collection Software v2.0 and DNA Sequencing Analysis Software v5.1.1, respectively (AB Applied Biosystems).

Pooled qPCR data from each experimental group (four animals per group and two technical replicates for each sample to make *n* = 8) were plotted as “mean intensity normalized” (MIN) ±standard deviation. The normalization was calculated as the percentage ratio with the housekeeping gene GAPDH. All data were exported to SigmaStat, 2.03^®^ (SPSS Inc^®^) for statistical analysis.

## 3 Results

### 3.1 Low frequency electrical stimulation of the sciatic nerve at C-fibre strength causes behavioural sensitization

**Fig. 1:**
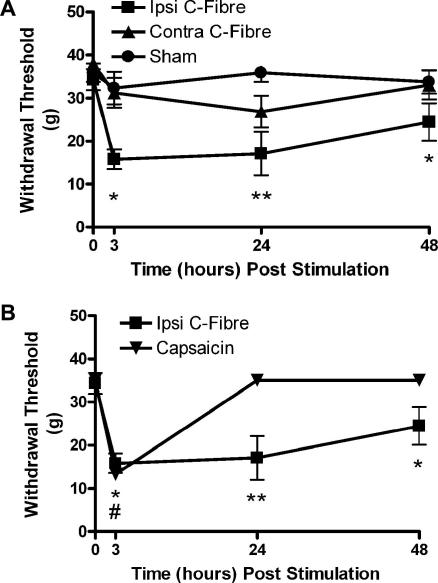
C-fibre strength electrical stimulation of the sciatic nerve induces a prolonged sensitization of mechanical withdrawal thresholds. (A) Thresholds were significantly decreased in the ipsilateral paw of stimulated rats when compared to sham animals and contralateral thresholds. (B) Activation of TRPV1 positive C-fibres with capsaicin also significantly reduced thresholds, however this was not as prolonged as that seen with electrical stimulation. Bars indicate mean ± SEM. Values are the means of 6–8 animals in each group. Asterisks indicate statistically significant differences between groups (two-way ANOVA with Bonferroni post-test) ^∗^*P* < 0.05, ^∗∗^*P* < 0.01 (electrical stimulation) ^#^*P* < 0.05 (capsaicin stimulation).

Electrical stimulation of the sciatic nerve at 10 Hz for 5 min at intensities sufficient to activate C-fibres (10 mA, 500 μs) resulted in a significant reduction in ipsilateral paw withdrawal thresholds to mechanical stimulation that lasted for over 48 h (Fig. 1A, ipsilateral C-fibre group vs sham; *P* < 0.05 (3 h) *P* < 0.01 (24 h), *P* < 0.05 (48 h) *n* = 8 per group). No significant changes in paw withdrawal thresholds were observed in sham-operated animals (Fig. 1A).

Unilateral subcutaneous injection of capsaicin into the plantar surface of the hindpaw also significantly decreased mechanical thresholds although this reduction was short-lived compared to that observed with C-fibre strength electrical stimulation of the sciatic nerve. The reduction in the capsaicin treated rats was only significant at the 3-h time point (Fig. 1B; *P* < 0.05, *n* = 6). By 24 h, mechanical thresholds had returned to baseline values. Mechanical withdrawal thresholds in the C-fibre stimulation group were significantly lower at this time point (24 h; *P* < 0.01, *n* = 6 and 8 capsaicin and C-fibre groups, two-way ANOVA).

### 3.2 Sciatic nerve stimulation at C-fibre, but not Aβ/Aδ, strength activates microglia in the dorsal horn

**Fig. 2:**
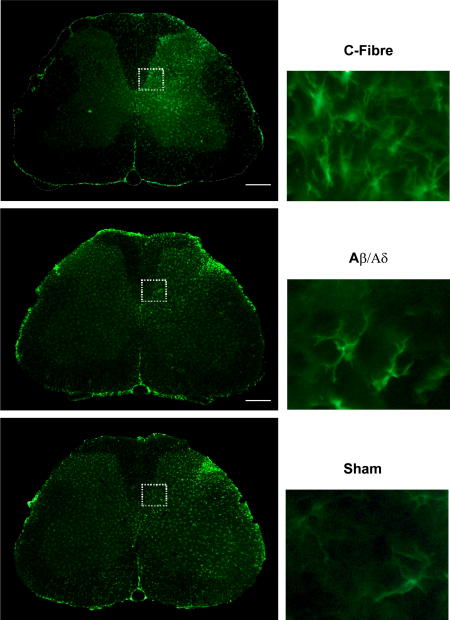
Electrical stimulation of the adult rat sciatic nerve is associated with changes in Iba-1 immunoreactivity in the L4 spinal dorsal horn. Expression patterns of Iba-1 immunoreactive microglia in the dorsal horn of rats that had undergone sham surgery or where the sciatic nerve was stimulated at Aβ/Aδ or C-fibre intensities 48 h previously. The greatest expression was in the lateral aspect of the dorsal horn for sham, and Aβ/Aδ groups however in the C-fibre group this area was in the medial aspect. Comparisons of the Iba-1 expression in this area are shown in the high power (X40) images that are displayed in the right hand column. Scale bars are 50 μm.

In separate groups of animals, lumbar spinal cord tissue was taken at 3, 24 and 48 h time points post-stimulation and was immunostained for Iba-1. The expression pattern of activated microglia in the dorsal horn was compared in sham, Aβ/Aδ, and C-fibre stimulated rats (Fig. 2). The pattern of Iba-1 expression in sham-operated and Aβ/Aδ group dorsal horn was indistinguishable. In both the groups, levels of Iba-1 expression were low except for a localized increase in Iba-1 immunoreactivity in the dorsal laminae of the ipsilateral side in the lateral L3/4 segments corresponding to the termination site of afferents damaged by the surgical incision to expose the sciatic nerve in the upper leg (Fig. 2). Examination of microglia within this region showed characteristic morphological changes and amoeboid cell bodies associated with microglial activation.

**Fig. 3:**
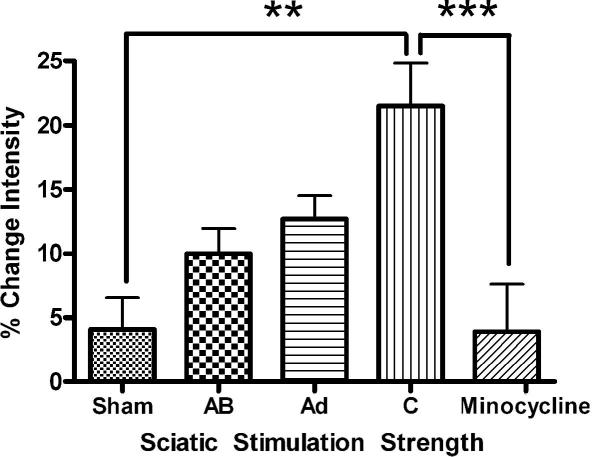
Quantification of the intensity of Iba-1 immunoreactivity in the medial aspect of the L4 dorsal horn of rats stimulated 48 h previously at increasing intensity shows clearly that increasing stimulus intensity leads to increased Iba-1 immunoreactivity. C-fibre strength stimulation leads to significantly greater Iba-1 immunoreactivity than in sham-operated spinal cord. Minocycline was able to prevent the increase in intensity seen with untreated c-fibre stimulated rats. Bars indicate mean ± SEM. Values are the means of 6–8 animals in each group. ^∗∗^*P* < 0.01, ^∗∗∗^*P* < 0.001.

The pattern of Iba-1 expression and the morphology of the microglia within the dorsal horn of the C-fibre group were quite different (Fig. 2). In this group the level of Iba-1 immunoreactivity in the ipsilateral dorsal horn was substantially increased particularly in the medial portion within the somatotopic area associated with sciatic nerve termination at the 24 h (data not shown) and 48 h time points. There were no differences in Iba-1 expression between the groups at the 3 h time point. This change was accompanied by altered microglial cell morphology to an amoeboid, swollen shape. Quantification of the Iba-1 immunoreactivity in the different stimulus groups at 48 h post-stimulation is shown in Fig. 3 which shows a significant difference in Iba-1 intensity between sham and C-fibre groups in the medial aspect of the dorsal horn (*P* < 0.05, one-way ANOVA). To allow for inter-animal variability the ipsilateral dorsal horn fluorescence intensity was calculated as a percentage of the contralateral value, although in the C-fibre group variable changes were observed in contralateral microglial morphology.

### 3.3 Sciatic nerve stimulation at C-fibre strength upregulates microglial markers in the dorsal horn

In order to quantify dorsal horn microglial activation following sciatic C-fibre stimulation, mRNA levels of markers of microglia activation were analysed using qPCR at 3, 24 and 48 h. Interleukin-6 (IL-6), monocyte chemo attractant protein 1 (MCP-1), metalloproteinase 3 (Mnp3) and the macrophage-specific colony-stimulator factor-1 (CSF-1) were used as markers of the initiation of microglial activation. Sustained microglial activation and migration were assessed by colony-stimulator factor-1 receptor (CSFR1), the macrophage scavenger receptor CD163 (which tags resident macrophages) and the macrophage marker of phagocytosis CD68 (which indicates the presence of infiltrating or activated macrophages).

The findings are summarised in Table 1 and show two phases of microglia activation. Firstly, there is a moderate but significant increase in the levels of compounds associated with the activation of microglia, IL-6 at 3 h post-stimulation, Mmp3 at 24 h and CSF-1 at both 24 and 48 h post C-fibre stimulation. In addition, the chemokine MCP-1 is increased at all three time points. The second phase is consistent with the maintenance of microglial activation and their migration to the affected tissue with upregulation of the markers CD163 and CSFR1 24 h post C-fibre stimulation. Interestingly, there were no differences in the mRNA levels of the macrophage infiltration CD68 over the 48 h when compared to the sham rats, suggesting that the activation following our stimulation protocol was restricted to resident microglia.

### 3.4 C-fibre stimulation-evoked mechanical hypersensitivity and microglial activation is prevented by minocycline treatment

**Fig. 4:**
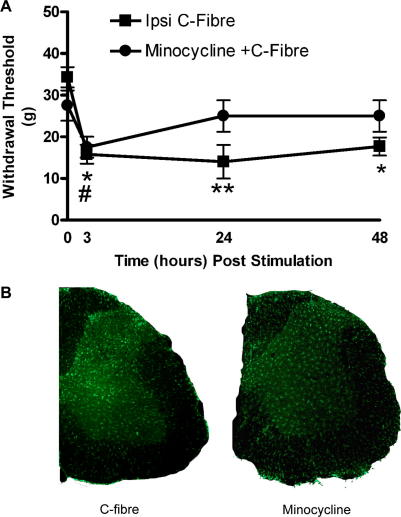
(A) Pre-treating rats prior to surgery with the microglial inhibitor minocycline (40 mg/kg, i.p.) and then every 24 h after surgery prevents the development of central sensitization associated with sciatic nerve stimulation at intensities that activate C-fibres. The reduction in mechanical withdrawal threshold in freely behaving rats at 24 h is prevented by pre-treating rats with minocycline (squares). (B) Minocycline also prevents the high intensity expression of Iba-1 in the medial portion of the dorsal horn of C-fibre stimulated rats. Bars indicate mean ± SEM. Values are the means of 6–8 animals in each group.^∗^*P* < 0.05, ^∗∗^*p* < 0.01, ^#^*p* < 0.05 minocycline group (Student *t*-Test).

Pre-treatment of rats with minocycline (40 mg/kg), a known inhibitor of microglial activation, prior to C-fibre strength electrical stimulation of the sciatic nerve and then every 24 h thereafter, significantly reduced the stimulation evoked behavioural sensitization at 3, 24 and 48 h. Fig. 4A shows that the mean fall in mechanical threshold, 24 h after sciatic C-fibre stimulation is prevented by minocycline treatment (*P* < 0.05, *n* = 8 in each group, *t*-Test).

Analysis of the spinal cord with Iba-1 immunoreactivity showed that minocycline treatment also prevented the increase in microglial activation normally caused by sciatic nerve C-fibre stimulation (Fig. 4B). Quantitative analysis of Iba-1 immunoreactivity in the L4-5 dorsal horn 48 h after C-fibre stimulation shows that is significantly reduced by minocycline treatment (Fig. 3 *P* < 0.001, one-way ANOVA).

These results demonstrate that brief low frequency C-fibre stimulation of the sciatic nerve leads to significant behavioural sensitization that persists beyond the stimulation period. Furthermore this sensitization is the result of microglial activation and can be prevented by pre-treating the rats with minocycline.

### 3.5 C-fibre electrical stimulation activates microglia and mechanical hypersensitivity in the absence of nerve pathology

**Fig. 5:**
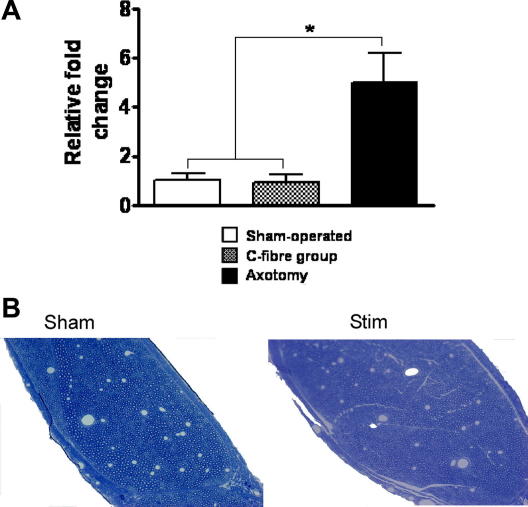
qPCR for ATF-3 a marker of neuronal damage shows no difference between sham and c-fibre stimulated rats, however in the positive control group (axotomy) levels of ATF-3 were significantly higher than those in either of the other two groups (B) Toluidine blue staining of the sciatic nerve shows no gross pathology of the sciatic nerve following stimulation. Bars indicate mean ± SEM. Values are the means of 6–8 animals in each group. ^∗^*P* < 0.05 (ANOVA, Bonferroni post-test).

A number of investigations were performed to assess any pathological changes to the sciatic nerve associated with electrical stimulation at C-fibre strength. Previous studies have determined that levels of Activating Transcription Factor 3 (ATF-3) significantly increase in the cell bodies of damaged primary afferent neurones [4,59]. However, qPCR quantification of ATF-3 mRNA in naive and C-fibre stimulated L4 and L5 dorsal root ganglia using polymerase chain reaction showed no significant differences in ATF-3 mRNA levels clearly indicating a lack of axonal damage in these two groups. This was in marked contrast to a sciatic axotomy group (positive control), where levels of ATF-3 mRNA were significantly increased (5- to 6-fold) in the ipsilateral affected L4/5 dorsal root ganglia relative to the contralateral side (Fig. 5A) reflecting the retrograde changes triggered in the cell bodies after axotomy.

Consistent with the lack of ATF3 expression, no differences were observed in the density or pattern of peptidergic (CGRP) and non-peptidergic C-fibre (IB4) primary afferent terminal staining in the ipsilateral dorsal horn of the C-fibre stimulation group (data not shown).

To further assess whether the C-fibre stimulation protocol caused direct damage to the sciatic nerve, microscopic examination of toluidine blue semi-thin sciatic nerve sections was performed in C-fibre and sham groups, 3 days after surgery (*n* = 3) (Fig. 5B). This clearly demonstrated the lack of any significant differences between sham-operated and electrically stimulated sciatic nerves. Detailed examination failed to find significant immune cell infiltration, axonal degeneration or abnormally swollen axons that are characteristic of nerve damaged tissue.

Together, these data clearly show that the behavioural changes associated with C-fibre strength sciatic nerve stimulation are not associated with pathological changes in the nerve.

### 3.6 C-fibre stimulation of neonatal sciatic nerve fails to induce behavioural hypersensitivity or to alter Iba-1 expression pattern

**Fig. 6:**
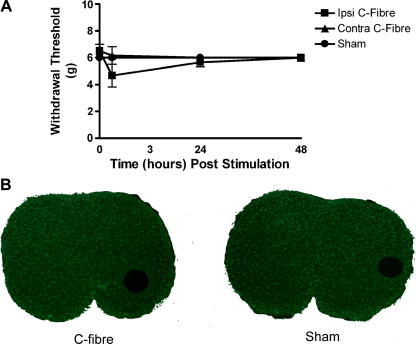
(A) Electrically stimulating the sciatic nerve of P10 rat pups fails to induce any behavioural sensitization in either the ipsilateral or the contralateral paw. There were also no changes in the expression pattern of Iba-1 immunoreactivity when compared to that in sham animals (B). Bars indicate mean ± SEM. Values are the means of 6–8 animals in each group.

Identical C-fibre and sham stimulation protocols were employed as with adult rats except in 10-day old (P10) rats. Basal, unstimulated, mechanical withdrawal thresholds were significantly lower than adult values, a phenomenon that has been widely described elsewhere [13]. Both C-fibre stimulated and sham animals failed to develop any significant mechanical hypersensitivity (Fig. 6A) following sciatic nerve stimulation. There were also no significant changes in the Iba-1 expression pattern in either of the groups. Interestingly this also extended to the lateral dorsal horn where the increase in Iba-1 immunoreactivity seen in the adult that is associated with the surgical incision was also absent in both the stimulated and sham groups (Fig. 6B). These results show that considerable postnatal modification of the immunological response to high frequency nerve activity takes place and that the lack of microglial activation may underpin the inability of neonatal rats to exhibit behavioural sensitization following neuropathic injury.

## 4 Discussion

This study provides compelling *in vivo* evidence that the microglial population of immunocompetent cells within the dorsal horn of the spinal cord can be activated by a brief period of peripheral nerve C-fibre stimulation, in the absence of pathology and this can lead to significant alterations in sensory processing within the dorsal horn. The stimulus-evoked changes in microglial activation were restricted to the somatotopic areas of the dorsal horn which receive sciatic nerve input and were sufficient to evoke significant and long-lasting changes in hindpaw mechanical sensitivity most likely due to central sensitization of nearby dorsal horn neurons. The hypersensitivity and alterations in Iba-1 expression pattern are not seen in neonatal rats identifying a potentially important maturational process that may underpin the inability of young animals to exhibit neuropathic pain behaviours following nerve injury.

One of the central characteristics of networks within the spinal dorsal horn is their ability to modify the efficacy of synaptic contact between the components of the network to either increase or decrease the amount of sensory information relayed on to upstream sites. This central sensitization is triggered by intense nociceptor stimulation, such as following tissue inflammation or nerve damage [20,21] or repetitive electrical C-fibre nociceptor stimulation [8,54,65] and leads to enhanced excitatory synaptic transmission in the dorsal horn [8,12]. Various mechanisms have been proposed to underlie central sensitization, including hetero- and homosynaptic potentiation, transcription-dependent changes in synaptic function and changes in the level of inhibition within the dorsal horn [19,34,38,46,47,50,56,57]. The changes in dorsal horn excitability which result from these processes can last from minutes to days and are thought to underpin chronic pain states [26,64].

Traditionally the interactions between the neuronal components of the pain pathway have been the focus of research on central sensitization, but more recently, interactions between neurons and immune cells have been the focus of interest, particularly microglia [22,36,52]. Microglia are derived from a hematopoietic heritage and make up to 5–10% of the total glial population in the CNS. Normally microglia are dormant, but once activated they undergo a series of changes in morphology, gene expression, function and number [44]. A causal link has been shown to exist between the activation of microglia and the onset of chronic pain following neuropathic lesions [23,58] and *in vivo* microinjection of exogenously activated microglia leads to behavioural sensitivity consistent with central sensitization [2,9,27,39].

To date the investigations into the involvement of endogenous microglia in experimental models of pain have always assumed that activation of these cells is dependent upon pathological modification of the tissue of interest. Microglia are analogous to macrophages and are known to play a role in the phagocytosis of cellular debris [41,43] Nerve damage, which triggers intense microglial activation, is accompanied by numerous injury-induced changes in the nerve stump, cell bodies and central terminals, including C-fibre ectopic firing and terminal atrophic changes [11,52] Here we have shown that significant changes in microglial distribution in the dorsal horn that are associated with central sensitization can occur without concomitant pathological modification of the peripheral innervation. The brief, low frequency C-fibre stimulation used here is a physiological rather than a pathological stimulus and we observed no abnormal nerve fibre profiles in ultra-thin sciatic nerve sections, no decrease in IB4 or CGRP terminals in the dorsal horn [17] and no increase in ATF-3, all sensitive markers of neuronal damage in the DRG [59] following this protocol. Electrical stimulation can cause nerve damage [37] but the protocol used here showed no evidence of this.

The behavioural sensitization we observed following electrical stimulation of the sciatic nerve was more prolonged than that observed with subcutaneous capsaicin, which selectively activates a population of C-fibres that express the TRPV1 receptor. This indicates that brief localized activation of cutaneous C-fibres alone is not sufficient to alter microglial markers in the dorsal horn, instead a critical number of C-fibres need to be stimulated or a critical duration of stimulation is required to cause microglial activation. The sciatic nerve is composed of a mixture of cutaneous and muscle afferents. It may be that activating some subpopulations of C-fibres is particularly effective in inducing microglial activation [65]. C-fibres are rapidly depolarised by topical application of capsaicin but this is paralleled by conduction block within a few minutes which will limit the C-fibre activation [33].

Minocycline is a tetracycline antibiotic that has been found to have numerous immunomodulatory activities. It inhibits the activity of matrix metalloproteinases, inducible nitric oxide synthase (iNOS), and cyclooxygenase-2 (COX-2) and impairs the production of cytokines such as Tumour necrosis factor-α (TNF-α) and interleukin (IL)-1β. Another critical target of minocycline is the inhibition of the proliferation and activation of microglia and the attenuation of T cell and microglial activation and microglial cytokine production and the down regulation of microglial MHCIII expression a [15,42]. Here we have concentrated on the effect of minocycline upon microglia but we make no assumptions about the mechanism by which this takes place. The ability of minocycline to prevent both the behavioural sensitization and the upregulation of Iba-1 immunoreactivity simply illustrates the central role that microglia play in the development of hyperalgesia. It has previously been shown that minocycline is able to prevent hyperalgesia when administered prior to either neuropathic or inflammatory insult [3,45,66], here we show that it can prevent C-fibre stimulation-evoked hyperalgesia and microglial activation.

Our data showing that neonatal rats do not become hyperalgesic and that the pattern of Iba-1 immunoreactivity does not change following electrical stimulation of the sciatic nerve at C-fibre intensities are consistent with the previous studies, which show that neonatal rats fail to express pain behaviours or activated microglia in the dorsal horn following neuropathic injury [18,39]. Rats do not express neuropathic pain until they are three weeks of age or older at the time of injury. Consistent with human data [1] we have previously shown that intrathecal injection of activated adult microglia to rats below P10 fails to cause behavioural hypersensitivity whereas they cause significant reductions in mechanical thresholds from P14 and in adult rats [39]. This current study indicates that P10 C-fibre activation is not capable of activating microglia or sensitizing behavioural responses.

Our results suggest that microglial activation and hyperalgesia are directly triggered by release of a factor(s) either from C-fibre terminals or from C-fibre activated postsynaptic cells in the dorsal horn. The time period of our electrical stimulation makes translational or transcriptional changes within the DRG or dorsal horn an unlikely contributor to this phenomenon. A plethora of compounds are available for release from the central terminals of C-fibres; glutamate, ATP, substance P, somatostatin and BDNF [6,29–31] as well as TNF-α and metalloproteinases [25] although these are unlikely to be in high enough concentrations in undamaged DRG. Our quantitative analysis of mRNA of markers of microglial activation in the dorsal horn following C-fibre stimulation suggests two phases of microglial activation. Compounds such as IL-6 are upregulated immediately following stimulation, provoking a microglial response. IL-6 has previously been shown to play a role in neuropathic pain and is known to cause microglial activation [67]. Mmp3 is a metallopeptidase that is known to cause degradation of major components of the extracellular matrix [53] and has recently been implicated inflammatory response [40]. The second phase corresponds to the migration of the activated microglia to the portion of the dorsal horn which has received the C-fibre input. This was demonstrated by the increase in MCP-1 (a chemokine involved in the recruitment of monocytes to sites of injury) and moreover MCP-1 is readily released from neurons [55] indicating a possible role in both the phases of the microglial response. The upregulation we observed with CSF-1 is again indicative of C-fibre evoked microglial activation whilst the increases in CD163 and absence of change in CD68 lead us to conclude that it is resident microglia that are activated following our stimulation paradigm. Further studies are required to study these pathways in more detail.

In conclusion we have shown that significant changes in sensory processing occur following the electrical activation of C-fibres in the sciatic nerve as a result of increased activation and altered distribution of microglia in the dorsal horn of the spinal cord. This is not the result of tissue damage but instead is the result of the release of compound(s) from primary afferent C-fibres.

## Figures and Tables

**Table 1:** Summary of qPCR. The table shows mean fold change in the gene expression ± SD. performed by comparison of the stimulated dorsal horn versus its counterpart sham at 3, 24 or 48 h post C-fibre stimulation. Mean fold changes are indicated (^∗^*P* < 0.05, power>80%).

Gene name	Time post stimulation		
	3 h	24 h	48 h
IL-6	1.3 ± 0.3^∗^	1.5 ± 0.8	1.0 ± 0.6
Mmp3	1.5 ± 0.7	1.8 ± 0.8^∗^	0.9 ± 0.4
Mcp-1	1.4 ± 0.2^∗^	1.3 ± 0.6^∗^	1.6 ± 0.4^∗^
CSF1	1.2 ± 0.6	1.3 ± 0.2^∗^	1.8 ± 0.5^∗^
CSFR1	1.0 ± 0.1	1 ± 0.4	1 ± 0.3
CD163	1.0 ± 0.1	1.7 ± 0 4^∗^	0.9 ± 0.1
CD68	0.9 ± 0.5	1.3 ± 0.3	0.9 ± 0.5
