# Supplementary material for: Brief, low frequency stimulation of rat peripheral C-fibres evokes prolonged microglial-induced central sensitization in adults but not in neonates
Source: Pain. 2009 Jul;144(1-2):110–8. doi: 10.1016/j.pain.2009.03.022 (PMC2702711; doi:10.1016/j.pain.2009.03.022)
Supplement: Supplementary data [file mmc1.doc]

Supplemental Table

[**Click here to download high resolution image**](http://ees.elsevier.com/pain/download.aspx?id=168790&guid=db072b5c-5a76-4432-9fb0-3eddc4a4d08f&scheme=1)

| lnterleukin-6 | Sense | CTTCC AGCC AGTT G C CTTCTTG |
| --- | --- | --- |
|  | Antisense | TGGTCTG TTG TG GGT G GTATCC |
| Chemokine (C-C motif) ligand 2 | Sense | ATGCAGTTAATGCCCCACTC |
|  | Antisense | TTCCT TAT TGGGGTC AG C AC |
| Matrix  metal lo peptidase 3 | Sense | TTG ATG AG AAG AAACAATCCATG |
|  | Antisense | CGCTG AAG AAG TAAAGAAA C C |
| Colony stimulating factor 1 (macrophage) | Sense | ATCCCGTTTG CTACCTAAAG |
|  | Antisense | AG CTG TTC AGTTTC ATAG AG AG |
| Colony stimulating factor 1 receptor | Sense | GTCATAC CT AC ATG TGC AAG G |
|  | Antisense | ATC CTCA CCAG CTTAGTAGG |
| ED1(CD68) | Sense | AAATACAAGC ATAGTTC TTTCTCC |
|  | Antisense | C A AG AG AG ATTG G TC AC TGG |
| ED2(CD163) | Sense | GTGTGTAAG CAACTGGGATG |
|  | Antisense | TCGC AGG AG AT ACTGTC AAG |
| Activating transcription factor 3 | Sense | AAGAGCTGAGATTCGCCATCC |
|  | Antisense | TCTCC AG AG G TCTGTTGTTG ATG |
| GAPDH | Sense | ACTCTAC CCACGGCAAGTTC |
|  | Antisense | GGTGGTG AAG AC G C CAG TAG |
